# Research Trends and Emerging Directions in Non-Pharmacological Interventions for Autism Spectrum Disorder: A Bibliometric Analysis (2001–2025)

**DOI:** 10.3390/healthcare14081108

**Published:** 2026-04-21

**Authors:** Yuting Lu, Wenliang Guo, Yanlin Zou, Ailing Wei, Jianwen Xu

**Affiliations:** 1 Department of Rehabilitation Medicine, The First Affiliated Hospital of Guangxi Medical University, Nanning 530021, China; 2 Department of Rehabilitation Medicine, Guangxi Medical University, Nanning 530021, China

**Keywords:** autism spectrum disorder, non-pharmacological interventions, bibliometrics, neuromodulation, rehabilitation therapy

## Abstract

**Highlights:**

**What are the main findings?**

- We mapped the evolution of non-pharmacological interventions for ASD over the period 2001–2025.
- Research has shifted from behavior-focused approaches to mechanism-informed studies, with emerging trends in neuromodulation and multimodal therapies.

**What are the implications of the main findings?**

- There is a growing emphasis on personalized interventions and enhanced clinical relevance in ASD research.
- Our findings provide evidence-informed priorities to guide and optimize future non-pharmacological intervention studies for ASD.

**Abstract:**

**Background**: Autism Spectrum Disorder (ASD) is a heterogeneous neurodevelopmental condition for which non-pharmacological interventions remain the primary therapeutic approach. Although research output in this field has increased substantially, a comprehensive synthesis of its developmental trajectory and emerging directions is still lacking. **Methods**: We conducted a bibliometric analysis of publications on non-pharmacological interventions for ASD indexed in the Web of Science Core Collection between 2001 and 2025. Knowledge structures, research hotspots, and temporal trends were visualized and analyzed using CiteSpace. **Results**: The field has transitioned from an early focus on behavioral interventions in children to a diversified and interdisciplinary research ecosystem spanning the lifespan. Recent growth has been driven by the integration of neuroscience-based approaches, particularly neuromodulation techniques, alongside continued refinement of behavioral, sensorimotor, and complementary therapies. Increasing attention has been paid to individual heterogeneity, methodological rigor, and mechanism-oriented research. Current frontiers emphasize multimodal intervention strategies, neural plasticity-based mechanisms, and the development of personalized precision intervention frameworks. **Conclusions**: This bibliometric analysis delineates the intellectual evolution of non-pharmacological intervention research for ASD and identifies key research gaps, particularly the need for longitudinal and pragmatic studies targeting individualized treatment response. The findings provide an evidence-informed overview of current concepts and emerging research directions in non-pharmacological care for ASD, with important implications for future clinical research, intervention design, and strategic research planning.

## 1. Introduction

Autism Spectrum Disorder (ASD) is a neurodevelopmental disorder with onset in early childhood, characterized primarily by impairments in social communication and interaction, as well as restricted interests and repetitive, stereotyped behaviors. The etiology of ASD is complex and is considered to result from the combined effects of genetic and environmental factors. The prevalence of ASD has shown a marked increase over the past few decades; however, most studies [1,2] suggest that this trend is largely attributed to non-biological factors—such as the broadening of diagnostic criteria, heightened public awareness, and improved screening and referral mechanisms—rather than a genuine surge in incidence.

In the absence of targeted pharmacological interventions for core symptoms, research and practice in non-pharmacological treatments have made remarkable progress over the past several decades, gradually evolving from a focus on focused behavioral training into a comprehensive system integrating neuroscience, rehabilitation medicine, psychology, and art therapy.

Neuromodulation techniques such as repetitive transcranial magnetic stimulation (rTMS) and transcranial direct current stimulation (tDCS), which target brain regions including the prefrontal cortex, have shown preliminary potential in improving social and executive functions. However, recent large-sample randomized controlled trials indicate that their efficacy is significantly influenced by individual differences and stimulation parameters. Future trends point toward personalized and combined intervention approaches [3,4].

In addition to neuromodulation, other intervention strategies continue to be refined and evaluated. Music therapy, while demonstrating promising findings in early studies, has not shown significant advantages in addressing core social impairments in large-scale randomized controlled trials, highlighting the importance of intervention format and individualized adaptation [5]. Sensory integration intervention, based on the widespread sensory processing abnormalities observed in ASD, aims to improve behavioral regulation and functional participation through structured sensory input. It has been recognized as an evidence-based practice, though further validation of its effectiveness in real-world settings is needed [6]. Furthermore, art therapy and animal-assisted therapy, as highly accepted complementary interventions, have shown potential in improving emotional regulation and social motivation [7]. Meanwhile, Early Intensive Behavioral Intervention is still regarded as the cornerstone for improving core functions, supported by the most robust long-term evidence base [8].

Collectively, the field is evolving from single-method approaches toward integrated intervention frameworks that are multimodal, personalized, and mechanism-informed.

Bibliometrics is an interdisciplinary methodology that integrates mathematics, statistics, and library and information science. It involves quantitative and qualitative analysis of literature data to reveal the research structure, developmental trajectory, and emerging trends within a specific field. The core of this approach lies in transforming scholarly outputs into measurable, comparable, and visualizable data, thereby enabling a macro-level understanding of the knowledge system. A typical bibliometric study comprises three fundamental steps: (1) systematic data retrieval from authoritative databases (e.g., Web of Science); (2) analysis using specialized tools (e.g., CiteSpace) for co-occurrence, co-citation, clustering, and burst detection; and (3) the preparation of an analytical report based on knowledge maps and statistical results to summarize the current landscape, identify research hotspots, and forecast future trends.

Bibliometrics has been widely applied across disciplines such as medicine, education, and psychology. However, within autism research, while bibliometric analyses have been conducted on sub-areas like neural mechanisms and diagnostic techniques, a systematic and comprehensive review focusing on non-pharmacological interventions as a key therapeutic framework is still lacking.

To address this gap, this study employs bibliometric methods to systematically map and analyze the literature on non-pharmacological interventions for autism. The objectives are to comprehensively delineate the field’s knowledge structure, collaboration networks, and developmental trajectories, and to identify core intervention strategies and emerging research directions. The findings are expected to provide an evidence-based foundation and inform clinical practice, research planning, and policy formulation.

## 2. Materials and Methods

### 2.1. Data Source and Search Strategy

This study employed a bibliometric approach to systematically review research literature on non-pharmacological interventions for autism spectrum disorder. The data were sourced from the Web of Science Core Collection, with the search conducted on March 15, 2026, covering the period from January 1, 2001, to December 31, 2025. The search strategy was developed based on preliminary searches and expert consultation, utilizing keywords as the search field. To balance comprehensiveness with precision, the final Boolean logic search formula was established as follows: ((TS = (autism OR autistic OR “Kanner syndrome” OR “Asperger syndrome” OR “pervasive developmental disorder”)) AND TS = (“behavioral intervention*” OR “behavioural intervention*” OR “joint attention” OR “social skill*” OR “parent-mediated” OR “transcranial magnetic stimulation” OR TMS OR rTMS OR “transcranial direct current stimulation” OR tDCS OR neuromodulation OR “sensory integration” OR “sensory-based” OR “music therapy” OR “art therapy” OR “animal-assisted” OR “equine-assisted” OR rehabilitation OR “physical activity” OR exercise)) NOT TS = (“stem cell*” OR “cell transplant*” OR pharmacological OR “drug therapy” OR medication OR supplement* OR oxytocin OR melatonin OR diet* OR “gene therapy”). The search language was restricted to English. No restrictions were applied to literature types during the retrieval phase to ensure a high recall rate; literature type screening was completed in subsequent steps.

### 2.2. Inclusion and Exclusion Criteria

Studies were included if they met all of the following criteria: (1) participants were individuals diagnosed with autism spectrum disorder (including Asperger syndrome, pervasive developmental disorder, etc.); (2) the research content fell within the scope of non-pharmacological interventions as defined by the search strategy, including behavioral interventions, neuromodulation, sensory interventions, art/music therapy, animal-assisted therapy, physical activity interventions, etc.; (3) the study type was original research articles providing primary empirical data, including randomized controlled trials, cohort studies, case series, etc.; (4) the study was published in English; (5) the study was published between January 1, 2001, and December 31, 2025. Studies were excluded if they met any of the following criteria: (1) non-English publications; (2) document types other than original research, including reviews, conference abstracts, editorial materials, proceedings papers, letters, book chapters, corrections, retracted articles, etc., as such document types are not suitable for co-occurrence analysis–based identification of research frontiers; (3) studies whose primary interventions fell under the categories of pharmacotherapy, stem cell therapy, dietary supplements, or gene therapy; (4) studies that did not focus on autism spectrum disorder or did not provide systematic evaluation of interventions, such as purely epidemiological investigations or diagnostic methodology studies.

### 2.3. Screening Process

**Figure 1:**
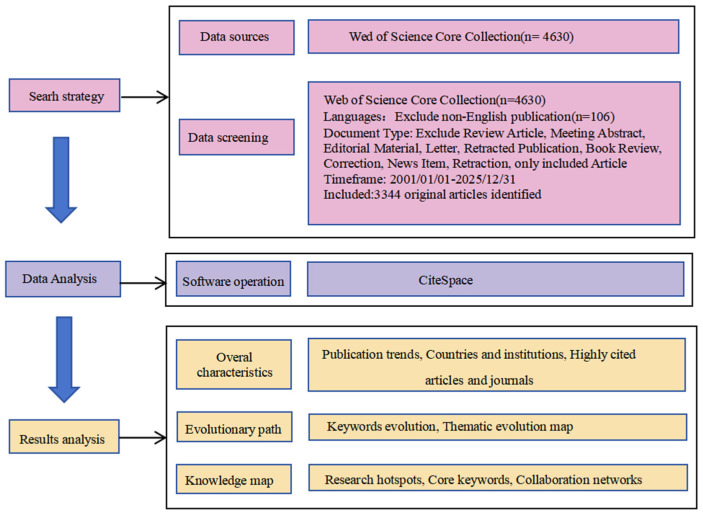
Research Flowchart.

The literature screening process is illustrated in Figure 1. The initial search yielded 4630 records. After excluding 106 non-English publications, 4524 records remained. Following further screening by document type, 1180 records classified as non-article types—including Review Article, Meeting Abstract, Editorial Material, Letter, Retracted Publication, Correction, Book Review, News Item, and Retraction—were removed, resulting in 3344 article records. Two researchers independently screened the titles and abstracts of these 3344 articles based on the predefined inclusion and exclusion criteria to assess their relevance. Disagreements regarding specific articles were resolved through discussion or consultation with a third researcher. Following manual review, all 3344 records met the inclusion criteria, and no articles were excluded. Ultimately, 3344 original research articles were included in the bibliometric analysis.

### 2.4. Bibliometric Analysis

This study employed CiteSpace 6.4.R1 for bibliometric analysis. CiteSpace is a Java-based application widely used to identify and analyze research trends and knowledge structures in scientific literature (CiteSpace II: Visualization and Knowledge Discovery in Bibliographic Databases).

The core parameters were configured as follows: the time span was set from January 2001 to December 2025, with a time slice length of one year. Node types were selected as author, country, institution, keyword, cited reference, and cited journal. For node selection, the primary analysis employed the g-index algorithm (k = 25), with the Top N = 50 approach used for sensitivity validation and the Top N% = 10.0% (with a maximum of 100 nodes per slice) applied as a supplementary threshold. Network pruning was performed using the Pathfinder algorithm for both slice networks and the merged network. Link strength was calculated based on cosine similarity, with the scope limited to “Within Slices”. Cluster analysis extracted labels using the log-likelihood ratio (LLR) algorithm. Burst detection was conducted using the algorithm embedded in CiteSpace, with the γ value set to the default of 1.0 to identify keywords and references with sudden increases in frequency. Betweenness centrality was used to measure the bridging role of nodes within the network structure; nodes with a centrality of ≥0.1 were considered key hubs. It should be noted that centrality reflects the structural importance of a node rather than directly representing scientific impact.

## 3. Results

### 3.1. Basic Quantitative Analysis

This study included 3344 research articles, originating from over 540 institutions in 82 countries. The included original studies encompass a variety of study designs, including randomized controlled trials, open-label trials, cohort studies, and case series, systematically reflecting the multidimensional development landscape of this field in both clinical practice and scientific research. The topics broadly cover various non-pharmacological interventions, such as behavioral interventions, neuromodulation techniques (e.g., TMS, tDCS), art and music therapy, animal-assisted therapy, sensory integration therapy, and acupuncture.

**Figure 2:**
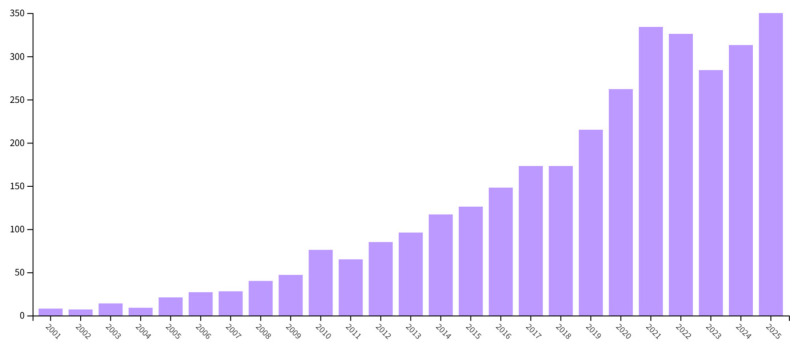
Annual Publication Trend.

Figure 2 illustrates the annual publication trend from 2001 to 2025, revealing distinct phases of growth. Overall, the field transitioned from a period of prolonged inactivity, through a phase of fluctuating growth, to a marked recent acceleration. Between 2001 and 2009, research activity was extremely limited, with annual publication counts remaining low (only 9 articles in 2004, increasing to 47 by 2009). Beginning in 2010, the number of publications showed a general upward trend, though with notable fluctuations; for instance, publications rose to 76 in 2010 but fell to 65 the following year. From 2012 to 2019, annual outputs steadily increased from 76 to 215 articles, indicating that the field was gaining attention and its momentum was continuously strengthening.

The year 2020 marked a critical turning point, with annual publications surpassing 200 (262 articles) for the first time, signaling the onset of a clear phase of rapid growth. Publications continued to rise thereafter, peaking at 338 articles in 2022. Subsequently, the annual publication counts remained at historically high levels, exceeding 280 articles in 2023 (284 articles), 2024 (313 articles), and 2025 (336 articles).

This trend indicates that, after nearly two decades of accumulation, the field has developed into a hotspot research direction characterized by high academic activity. The sustained high output since 2020 reflects both heightened scholarly attention and consistent investment from the academic community.

### 3.2. International Collaboration and Distribution of Research Strengths

**Figure 3:**
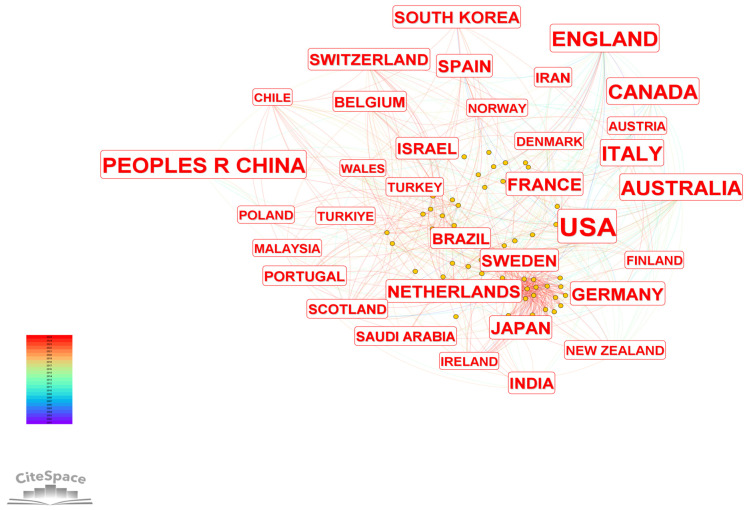
Country/Region Collaboration Network Map.

Figure 3 shows the characteristics of national/regional collaboration networks in this research field from 2001 to 2025.The frequency (number of publications) and betweenness centrality data of the relevant core nodes are shown in Table 1. The analysis reveals a global collaboration landscape dominated by a few core countries, characterized by broad participation and differentiated roles among nations. The United States ranks as the largest knowledge producer, with 1305 publications. Its betweenness centrality of 0.23 indicates that it plays a critical radiating and structural connecting role in the global collaboration network, serving as a core hub node. China follows with 389 publications, indicating a substantial research scale; however, its lower betweenness centrality (0.02) suggests that its bridging role in global cooperation still has considerable room for improvement. England (322 publications, 0.17) and Australia (250 publications, 0.14) rank among the top in terms of publication volume and both exhibit high betweenness centrality, serving as important structural hubs second only to the United States. They play a key role in connecting research clusters across different regions and facilitating cross-regional knowledge flow.

Regarding the timeline of participation, countries such as the United States, England, Italy, and France have been involved in this field since 2001, representing early pioneers. China, Canada, and Australia successively joined the research community between 2003 and 2004, becoming core forces in driving the expansion of research scale in this field. Germany, Japan, and Spain gradually became involved between 2005 and 2006, further diversifying the global research landscape and fostering a pattern of multinational collaborative research.

In summary, the collaboration network exhibits a typical core–periphery structure, forming a complex cooperation system with the United States as the core hub, England and Australia as key structural nodes, China as a major contributor in terms of research output, and extensive participation from multiple countries. This structure not only relies on core countries to achieve efficient knowledge production and frontier exploration in the field, but also promotes the globalization of research through the involvement of multiple countries and regions. However, the limited network connectivity of some major producing countries represents an important area for optimization in the current development of the global collaboration network.

### 3.3. Analysis of Core Research Institutions

**Figure 4:**
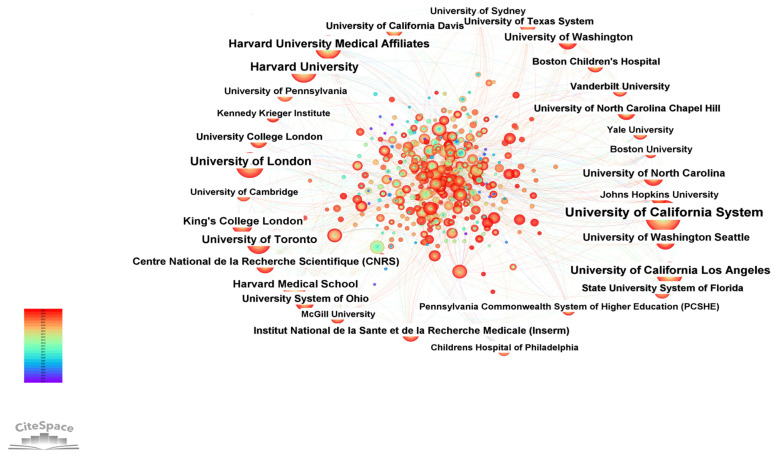
Collaboration Network Map of Research Institutions in Autism Spectrum Disorder (ASD) Non-pharmacological Intervention Studies.

Figure 4 and Table 2 present the institutional collaboration network structure within this field, whose key nodes consist of a group of high-output and high-impact leading academic and medical centers. In terms of research output (frequency), the University of California System (200 publications), University of London (129 publications) and Harvard University (117 publications) are the three core institutions with the highest publication counts, forming the top tier of global knowledge production. Harvard-affiliated medical institutions and the University of California, Los Angeles (UCLA) are tied for fourth place, each with 98 publications. Institutions such as the University of Toronto (90 publications) and Harvard Medical School (79 publications) also demonstrated notable research output. 

In terms of institutional types, the core of the network encompasses three categories of entities. The first category consists of world-leading comprehensive research universities, represented by the University of California system, the University of London, and Harvard University, which serve as the central hubs of knowledge production in the field. The second category comprises high-level affiliated medical institutions, exemplified by Harvard-affiliated medical institutions and Harvard Medical School, which act as crucial bridges facilitating the translation of basic research into clinical practice. The third category includes national-level research institutes, such as the French National Centre for Scientific Research (CNRS) and the French National Institute of Health and Medical Research (INSERM), which provide important strategic scientific support for foundational theoretical research in the field. These three types of institutions operate synergistically, forming a diversified research landscape characterized by a “basic research–clinical translation–strategic support” framework.

From a temporal perspective, the University of California system and the French National Institute of Health and Medical Research (INSERM) have been involved in this field since 2001, while the University of London, the University of California, Los Angeles (UCLA), and King’s College London successively began their research contributions in 2002, positioning them as early pioneers and founders of the institutional collaboration network in this field. Harvard University, Harvard-affiliated medical institutions, and Harvard Medical School gradually emerged as key research drivers between 2006 and 2008, accelerating the rapid expansion of research scale in the field. Institutions such as the University of Toronto, the University of North Carolina, and the University of Washington joined the core research cohort between 2010 and 2011, further enriching the hierarchy and scope of the institutional collaboration network and continuously injecting research momentum into the network. 

### 3.4. Highly Cited Publications and Core Journals Distribution

The analysis of highly cited publications and highly cited journals (Table 3 and Table 4) further reflects the research landscape in the field of non-pharmacological interventions for autism from the perspectives of knowledge base and core dissemination channels. Table 3 shows that there are 10 highly cited publications in this field, with citation frequencies ranging from 39 to 151. Among them, the study by Carter and colleagues published in Therapeutic Recreation Journal in 2014 has the highest citation count (151), while the majority of the other highly cited works were published between 2015 and 2023. In terms of document types, these include epidemiological reports published in MMWR Surveillance Summaries, such as those by Baio in 2018 and by Maenner in 2021 and 2023. Diagnostic and assessment instruments are represented by the Vineland Adaptive Behavior Scales developed by Sparrow and colleagues in 2016. Intervention meta-analyses are represented by the work published by Sandbank and colleagues in Psychological Bulletin in 2020. Authoritative reviews include those published by Lord and colleagues in Lancet in 2018 and in Nature Reviews Disease Primers in 2020. Research on early intervention models is represented by the work published by Schreibman and colleagues in the Journal of Autism and Developmental Disorders in 2015. Notably, all highly cited publications have a centrality of 0, indicating that while they exert substantial influence, they function primarily as foundational research rather than key bridging nodes within the knowledge network. Table 4 presents the highly cited journals in the field. Among the top 10 most cited journals, the Journal of Autism and Developmental Disorders ranks first with 2709 citations, followed by Autism (1561), the Journal of Child Psychology and Psychiatry (1434), and Autism Research (1257). In terms of disciplinary distribution, the highly cited journals are concentrated in autism-specific journals, developmental and child psychiatry journals, and multidisciplinary journals (e.g., PLoS ONE), reflecting the core channels of knowledge dissemination and the interdisciplinary nature of the field. All highly cited journals also exhibit a centrality of 0, suggesting their consistent foundational role as stable knowledge carriers within the document co-citation network.

### 3.5. Keyword Analysis

#### 3.5.1. Keyword Co-Occurrence

**Figure 5:**
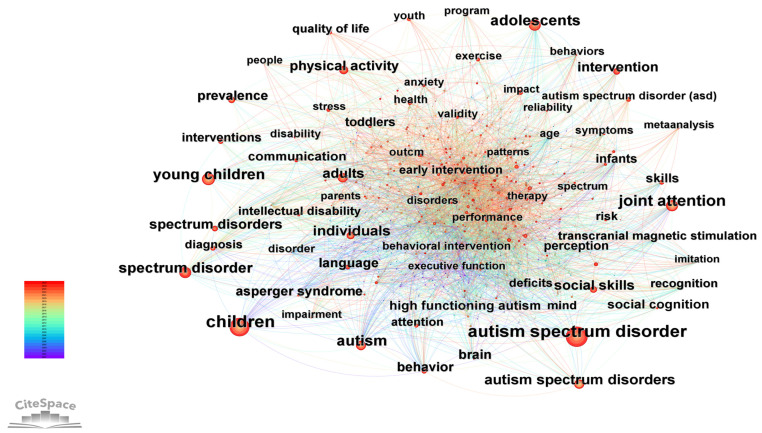
Keyword Co-Occurrence Network Map in ASD Non-Pharmacological Intervention Research.

Through co-occurrence analysis of keywords extracted from a large body of research, high-frequency terms were categorized and their association strengths evaluated, thereby revealing the intrinsic knowledge structure and research frontiers in this field (Figure 5, Table 5). The evolution of the knowledge structure from 2001 to 2025 exhibits a clear developmental trajectory with distinct phase characteristics. The research network exhibits distinct characteristics, including a stable core framework, a progressively expanding scope of research subjects, and a deepening of research themes from foundational characterization toward mechanisms and interventions. The core foundation of the research network is jointly constructed by keywords related to disorder definition and core study populations, serving as the cornerstone throughout the field. Among these, children (frequency: 955, betweenness centrality: 0.03) is the most frequent core node, closely followed by autism spectrum disorder (frequency: 899, betweenness centrality: 0.03). Together with autism (frequency: 443, betweenness centrality: 0.06), these three constitute the core framework of the research network. Notably, deficits (frequency: 97, betweenness centrality: 0.1) has the highest betweenness centrality among all keywords in the table, functioning as a key hub connecting various symptoms, disorders, and intervention studies, highlighting that the “identification and remediation of deficits” represents a central research logic in this field. From the perspective of core conceptual evolution, the foundational concept autism emerged in 2001, followed by the appearance of autism spectrum disorder in 2003, which subsequently became the dominant term. Between 2005 and 2006, derivative keywords such as spectrum disorder and autism spectrum disorders appeared with high frequency, reflecting the scholarly community’s deepening understanding of the heterogeneous nature of the autism spectrum and the progressive refinement of research definitions and scope.

The expansion of research subjects demonstrates a clear trend toward full lifecycle coverage, progressively extending from a focus on specific age groups to the entire population, accompanied by the continuous refinement of subpopulation studies. In 2001, research initially focused on two core populations: children and adults (frequency: 222, betweenness centrality: 0.05). Subsequently, keywords such as infants (frequency: 125, betweenness centrality: 0.03) appeared in 2002, young children (frequency: 442, betweenness centrality: 0.01) in 2003, adolescents (frequency: 402, betweenness centrality: 0.03) in 2005, and toddlers (frequency: 116, betweenness centrality: 0.02) in 2011. Meanwhile, individuals (frequency: 296, betweenness centrality: 0.02) emerged as a high-frequency keyword, reflecting a shift in research from “specific age groups” toward full lifecycle coverage encompassing “young children–children–adolescents–adults.” Furthermore, attention to subgroups such as high functioning autism (frequency: 148, betweenness centrality: 0.03) further reflects the refinement and stratification of research subjects, aligning with the field’s deepening exploration of disorder heterogeneity.

The research themes exhibit a systematic progression over time, evolving from foundational characterization and diagnosis toward symptom mechanisms, intervention strategies, and functional improvement, thereby forming a multidimensional research system with strong interconnections among themes. In the early foundational research phase (2001–2004), the focus was on core symptoms of the disorder, basic cognition, and the construction of diagnostic frameworks. Keywords such as joint attention (frequency: 509, betweenness centrality: 0.02), attention (frequency: 101, betweenness centrality: 0.03), behavior (frequency: 166, betweenness centrality: 0.05), and brain (frequency: 172, betweenness centrality: 0.03) emerged as hotspots. The appearance of diagnosis (frequency: 98, betweenness centrality: 0.03) and Asperger syndrome (frequency: 169, betweenness centrality: 0.04) marked an extension from symptom identification toward diagnostic subtyping and exploration of pathological foundations. The early emergence of early intervention (frequency: 93, betweenness centrality: 0.03) also established “intervention as a core focus” as a central research priority.

During the theme expansion and refinement phase (2005–2009), research extended toward symptom subclassification, social functioning, and perceptual mechanisms. Keywords such as social skills (frequency: 236, betweenness centrality: 0.02), communication (frequency: 148, betweenness centrality: 0.02), perception (frequency: 116, betweenness centrality: 0.03), and behaviors (frequency: 86, betweenness centrality: 0.05) emerged with high frequency. Concurrently, studies on prevalence (frequency: 190, betweenness centrality: 0.01) and intellectual disability (frequency: 105, betweenness centrality: 0.02) advanced the understanding of epidemiological patterns and comorbid characteristics in the field. The emergence of transcranial magnetic stimulation (frequency: 108, betweenness centrality: 0.03) marked the beginning of neuromodulation techniques as a new direction in intervention research, signaling the extension of research paradigms toward biomedical interventions.

Intervention Deepening and Functional Improvement Phase (2010 onward): Research focused on optimizing intervention strategies, exploring social cognitive mechanisms, and addressing long-term prognosis. Interventions (frequency: 132, betweenness centrality: 0.02) emerged as a hotspot, replacing foundational concepts and reflecting the diversification of intervention approaches. Studies on social cognition (frequency: 137, betweenness centrality: 0.01) and recognition (frequency: 101, betweenness centrality: 0.03) advanced in-depth exploration of symptom mechanisms. The emergence of keywords such as physical activity (frequency: 235, betweenness centrality: 0.01), health (frequency: 88, betweenness centrality: 0.03), and quality of life (frequency: 91, betweenness centrality: 0.01) indicated a shift in research emphasis from “symptom alleviation” toward “functional enhancement and optimization of long-term prognosis.” Meanwhile, research on risk (frequency: 99, betweenness centrality: 0.01) further highlighted the field’s trend toward precision and forward-looking approaches.

Furthermore, the betweenness centrality characteristics of keywords also reflect the connective value of research themes within the network. Keywords such as behavior, behaviors, and adults exhibit both high frequency and relatively high betweenness centrality, serving as important nodes that connect symptom research, population studies, and intervention research. Emerging research directions such as transcranial magnetic stimulation and high functioning autism demonstrate moderate levels of betweenness centrality, indicating that they have established connections with core research themes in the field and are gradually becoming important branches within the network.

#### 3.5.2. Keyword Clustering

**Figure 6:**
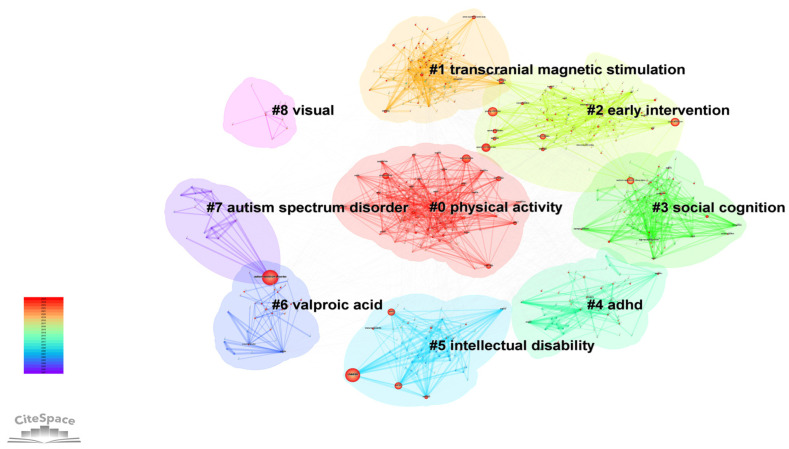
Keyword Cluster Map of ASD Non-Pharmacological Intervention Research.

In this study, a keyword co-occurrence and cluster analysis was conducted using CiteSpace on research in the field of non-pharmacological interventions for autism from 2001 to 2025. A total of 682 nodes and 3810 links were included, with a network density of 0.0164. The largest connected component accounted for 98.53% of the network, indicating a high degree of interconnectedness and concentration within the research field. Cluster analysis identified eight major research themes, with a modularity Q value of 0.3859 and a weighted mean silhouette S value of 0.6691, suggesting significant cluster structure and high internal homogeneity. As shown in Figure 6,. the specific clusters are as follows: #0 physical activity, #1 transcranial magnetic stimulation, #2 early intervention, #3 social cognition, #4 attention-deficit/hyperactivity disorder (ADHD), #5 intellectual disability, #6 valproic acid, #7 autism spectrum disorder, and #8 visual. The above clusters reflect that the core research themes in this field are primarily concentrated on early behavioral interventions, neuromodulation techniques, exercise rehabilitation, comorbidity management, and basic mechanistic research. These themes are closely interconnected, collectively forming the knowledge structure system of non-pharmacological interventions for autism.

#### 3.5.3. Keyword Burst Detection

**Figure 7:**
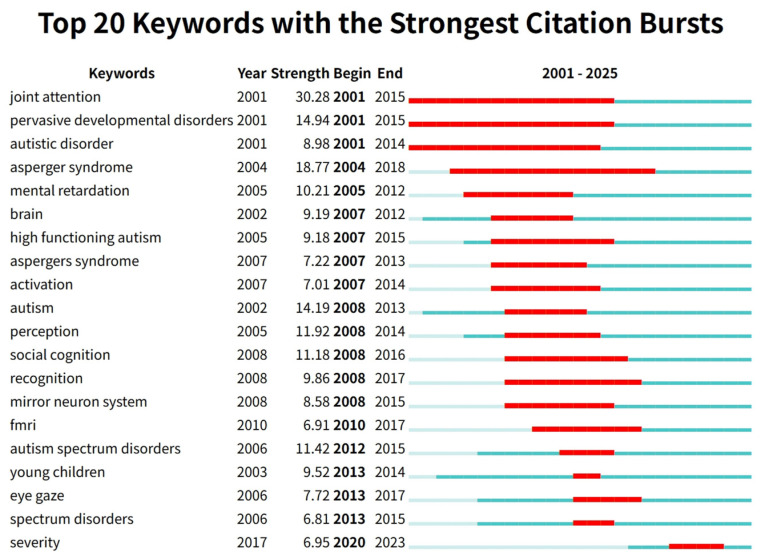
Keyword Burst Detection Evolution Map of ASD Non-Pharmacological Intervention Research. The blue line represents the time intervals, while the red line indicates the periods of significant keyword bursts.

Keyword burst detection analysis is a powerful tool for identifying research frontiers and predicting future trends within a discipline. It identifies keywords whose frequency of occurrence undergoes a sharp, statistically significant increase within a specific time period. The “burst strength” reflects the intensity of attention a hotspot receives, while the “start-end years” define its active period, thereby revealing the evolving research frontiers across different stages. This study employed CiteSpace to perform keyword burst detection. The results clearly delineate the research evolution over the past two decades into three distinct phases (see Figure 7). In the early period (2001–2007), keywords with relatively high burst strength primarily focused on clinical diagnosis and basic phenotypes, such as joint attention (burst strength 30.28, burst period 2001–2015), pervasive developmental disorders (burst strength 14.94, burst period 2001–2015), autistic disorder (burst strength 8.98, burst period 2001–2014), and Asperger syndrome (burst strength 18.77, burst period 2004–2018), indicating that research priorities during this phase centered on core symptom identification and the construction of classification systems. In the middle period (2007–2017), burst keywords progressively expanded toward neural mechanisms and cognitive processing, including brain (2007–2012), activation (2007–2014), mirror neuron system (2008–2015), fMRI (2010–2017), as well as social cognition (2008–2016) and recognition (2008–2017), reflecting the widespread application of neuroimaging and cognitive neuroscience methods in the field. In the recent period (2013–2023), burst keywords reveal a shift toward early intervention, clinical assessment, and attention to individual differences, such as young children (2013–2014), eye gaze (2013–2017), and severity (2020–2023). Notably, severity exhibited a sustained burst from 2020 to 2023, suggesting that recent research has increasingly focused on the heterogeneity of symptom severity and individualized intervention strategies. Overall, the research hotspots in this field have evolved along a trajectory from “diagnostic classification” to “neural mechanisms” and then to “individualized assessment and intervention”.

#### 3.5.4. Timeline Visualization

**Figure 8:**
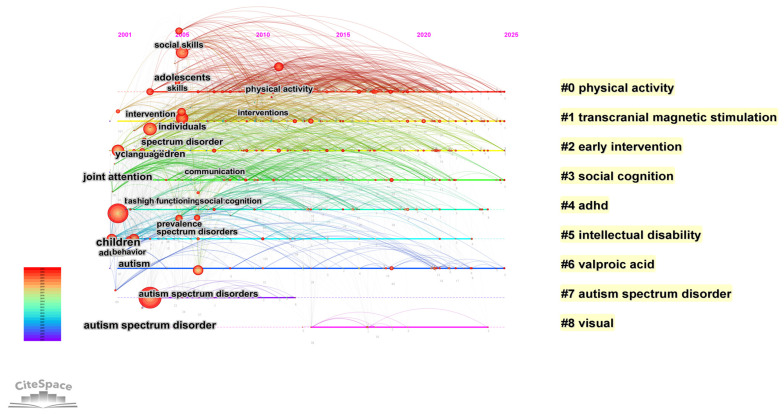
Timeline of Research Theme Evolution in ASD Non-Pharmacological Intervention Studies.

Based on the timeline visualization generated by CiteSpace, we were able to visually track the emergence, evolutionary trajectories, and interrelationships of distinct research themes between 2001 and 2025 (Figure 8). This visualization maps the results of the cluster analysis along a temporal axis, where the Y-axis represents different research theme clusters (e.g., #0 to #8), the X-axis denotes time, node size corresponds to keyword frequency, and connecting lines indicate co-occurrence relationships. This presentation not only helps identify persistently active foundational themes but also clearly reveals emerging frontier directions.

The knowledge architecture presents a multi-level, cross-paradigm, and logically coherent research system. These clusters do not exist in isolation; instead, they collectively outline a complete knowledge landscape spanning from macro-level diagnostic frameworks to micro-level neural mechanisms, and from diverse intervention approaches to focused attention on specific populations.

In terms of the overall trend, early research was primarily concentrated in Cluster #2 early intervention and Cluster #7 autism spectrum disorder, with high-frequency nodes such as joint attention, communication, and pervasive developmental disorders peaking around 2001–2010, indicating that this phase was dominated by core symptom identification and early behavioral interventions. After 2010, Cluster #0 physical activity and Cluster #3 social cognition gradually became research focal points, with nodes such as social skills, adolescents, and high functioning autism remaining active until approximately 2020, suggesting that social cognition training and intervention research targeting adolescent populations were gaining increasing attention. Meanwhile, Cluster #1 transcranial magnetic stimulation showed a marked increase in node density from 2015 onward and formed connections with Cluster #6 valproic acid, reflecting the rapid emergence of neuromodulation techniques and mechanistic studies using animal models over the past decade. Cluster #4 ADHD and Cluster #5 intellectual disability spanned the entire research period, reflecting sustained attention to comorbid conditions in autism. Overall, the timeline visualization indicates that the research frontiers in this field have expanded from an early focus on behavioral interventions to encompass multiple directions, including social cognition training, neuromodulation techniques, and comorbidity management, exhibiting an evolutionary pattern characterized by the parallel development and cross-integration of multiple themes.

## 4. Discussion

ASD is a complex neurodevelopmental disorder characterized by core symptoms including social communication deficits, repetitive and stereotyped behaviors, and restricted interests. While no curative treatment exists, diverse non-pharmacological interventions play a crucial role in ameliorating core symptoms, enhancing functional levels, and improving quality of life. This study employed bibliometric analysis to systematically examine the knowledge structure and developmental trajectory of the field of non-pharmacological interventions for ASD from 2001 to 2025. From the perspective of annual publication trends, research interest in ASD has steadily increased over the past two decades. Particularly after 2010, with the integration of neuroimaging, brain stimulation techniques, and precision medicine concepts, research themes have experienced explosive growth. Among them, “children” (955 occurrences) and “autism spectrum disorder” (899 occurrences) are the most central keywords, reflecting the focus of research subjects and themes. Meanwhile, the emergence of keywords such as “physical activity” (235 occurrences, first appeared in 2011), “machine learning” (20 occurrences, first appeared in 2020), and “tdcs” (3 occurrences, first appeared in 2024) signals a paradigm shift from traditional behavioral observation toward an interdisciplinary, technology-driven comprehensive research model.

The bibliometric analysis in this study reveals that the dynamic evolution of research hotspots over time closely aligns with the actual developmental trajectory of non-pharmacological intervention research for ASD over the past two decades. Research in this field has evolved from an early focus on behavioral interventions and diagnosis, to a mid-term expansion into neural mechanisms and brain stimulation techniques, and more recently to the integration of artificial intelligence, physical activity interventions, and individualized treatment. The United States, China, and the United Kingdom are the core countries in this research area, while institutions such as the University of California system and the University of London serve as the main drivers of knowledge production. The early research network was centered on “ASD” and “children,” reflecting the field’s initial focus on defining the disorder and its application within the primary affected population. Research during this period focused on diagnostic subtyping (e.g., “Asperger syndrome”), basic behavioral interventions, and core manifestations (e.g., “anxiety”). This aligns with the initial phase of disciplinary development, characterized by clarifying the subject of study and exploring foundational intervention approaches [9]. Following the 2010s, a sharp rise in the centrality of keywords represented by “rTMS” marked a profound paradigm shift in research. This shift did not occur in isolation; rather, it stemmed from the concurrent deepening of neuroscience-based understanding of ASD pathological mechanisms, particularly the proposed hypotheses of cortical excitation/inhibition imbalance and specific neural circuit dysfunctions [10]. A substantial body of research has subsequently applied tools such as rTMS to directly modulate target brain regions, including the dorsolateral prefrontal cortex, in an attempt to alleviate core symptoms such as executive dysfunction and repetitive behaviors, thus promoting a shift from purely behavioral observation toward neurobiologically informed intervention [11]. The emergence of keywords such as “tDCS” and “theta burst stimulation,” alongside the prominence of the methodological label “randomized controlled trials,” collectively delineate two distinctive features of current research: first, the ongoing diversification and refinement of neuromodulation techniques [12]; second, the pursuit of higher evidence levels and scientific rigor has reached an unprecedented standard [13].

Research efforts in this field exhibit clear characteristics of globalization and multi-center collaboration. The United States (1305 publications) dominates research in this field by a substantial margin, and its high centrality (0.23) further confirms its role as a core hub within the knowledge network. China ranks second in terms of publication volume (389 publications), yet its relatively low centrality (0.02) suggests that its research is predominantly produced independently, with considerable room for improvement in its bridging role in international collaboration and knowledge dissemination. Countries such as the United Kingdom (322 publications), Italy (282 publications), and Canada (255 publications) also play important roles. At the institutional level, the University of California system (200 publications), the University of London (129 publications), and Harvard University (117 publications) are the major academic centers. Their output is not only substantial in volume but also leads global research directions in terms of methodology and theoretical frameworks. For instance, large-scale randomized controlled trials and a series of mechanistic studies on rTMS have been predominantly led by academic medical centers in the United States (e.g., teams led by Casanova and Sokhadze), Canada (e.g., teams led by Ameis and Daskalakis), and Australia (e.g., teams led by Enticott and Fitzgerald) [3,14,15,16,17]. The large-scale multicenter trial TIME-A on music therapy encompassed collaboration among teams from multiple European countries [5]. Regarding Sensory Integration Therapy, high-quality randomized controlled trials have also been conducted in regions such as Brazil and Thailand, contributing to the evidence base for evidence-based practice [18]. These patterns of international collaboration and regional variations in research activity are influenced by factors such as national research investment and healthcare systems. They also reflect that ASD intervention is a health issue of shared global concern. The formation of highly productive authors and collaboration networks often centers around specific intervention technologies or theoretical schools. For example, closely knit academic communities have formed around neuromodulation, music therapy, or early behavioral intervention, accelerating the accumulation and dissemination of knowledge.

**Figure 9:**
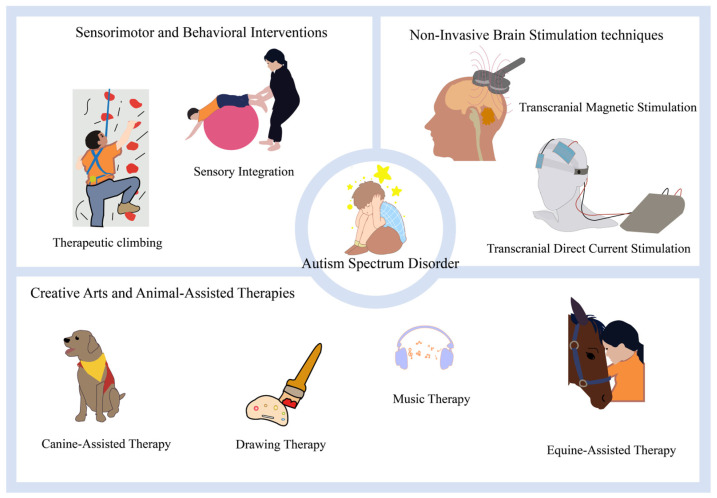
Schematic Diagram of Non-Pharmacological Interventions for Autism.

Based on keyword co-occurrence and cluster analysis, this study identified three core research pathways within the field of ASD non-pharmacological intervention that are both independent and interrelated (Figure 9).

First is the pathway of the rise of neuromodulation technologies and mechanistic exploration. Non-invasive brain stimulation techniques, particularly rTMS and tDCS, have emerged as one of the most promising biological intervention directions in this field due to their ability to directly and non-invasively modulate neural activity in brain regions implicated in ASD [19,20]. In bibliometric analyses, the emergence of keywords such as “transcranial magnetic stimulation” (108 occurrences, 2009) and “transcranial direct current stimulation” (19 occurrences, 2015), along with more recent terms like “theta burst stimulation” (11 occurrences, 2023), indicates continued growth in research interest in this area. Its developmental trajectory clearly reflects a progression from the initial introduction of the technology to the subsequent deepening of mechanistic understanding. It has progressed from proof-of-concept studies based on the cortical excitation/inhibition imbalance theory [10], to preliminary exploratory open-label trials [21,22] and efficacy confirmation via randomized controlled trials [23], and is currently advancing toward a new phase focused on stimulation protocol optimization and personalized precision intervention [12,24]. Studies have employed techniques such as electroencephalography, functional magnetic resonance imaging, and magnetic resonance spectroscopy to investigate its mechanisms of action, including the modulation of gamma oscillations, alterations in brain network connectivity, and the effects on glutamatergic neurotransmission [25,26]. The current forefront focuses on individualized target localization (such as that based on resting-state functional connectivity) [27], parameter optimization, and integration with cognitive training, aiming to achieve precise neuromodulation [4]. Systematic reviews and network meta-analyses have integrated the evidence for this pathway, indicating moderate effect sizes and an overall favorable safety profile, although optimal protocols remain to be determined [24,28]. Future clinical trials, particularly those investigating neuromodulation and complementary therapies, must adopt large-sample, randomized, double-blind, sham/placebo-controlled designs with pre-registration to minimize bias. Outcome measures should be standardized and focus on clinically meaningful changes rather than solely on statistical differences in scale scores. Systematic reviews and meta-analyses should adhere to the PRISMA guidelines and emphasize the assessment of evidence quality (e.g., using the GRADE system).

The second core pathway is the evidence-based refinement of sensorimotor and behavioral interventions. Behavioral interventions (e.g., applied behavior analysis, Early Start Denver Model) have the strongest evidence base and represent the gold standard in clinical practice. Behavioral interventions, particularly early intensive behavioral interventions grounded in applied behavior analysis principles, have long been considered the “gold standard” for non-pharmacological treatment of autism spectrum disorder. This perspective is well reflected in the bibliometric data: keywords such as “behavior” (166 occurrences, 2002), “early intervention” (93 occurrences, 2002), and “behavioral intervention” (75 occurrences, 2010) exhibit high frequencies and early emergence years, indicating that behavioral intervention has consistently remained a core focus of research in this field. Interventions targeting the sensory and motor systems have long been an essential component of its non-pharmacological treatment. The research focus is now shifting from efficacy verification toward elucidating underlying mechanisms and standardizing protocols. This pathway encompasses classic and persistently active keywords such as “sensory integration,” “music therapy,” “behavioral intervention,” “early intensive behavioral intervention,” and “naturalistic developmental behavioral interventions.” Sensory integration theory and physical activity interventions occupy a unique position in the non-pharmacological treatment of autism spectrum disorder. Bibliometric data reveal the following: the emergence of keywords such as “sensory integration” (42 occurrences, 2010), “motor skills” (45 occurrences, 2019), and “postural control” (19 occurrences, 2021), along with the high citation frequency of A. Jean Ayres’ classic work [29], indicates that both the theoretical foundation and practical application of this area are extensive. Sensory Integration Therapy, based on Ayres’ theory, aims to enhance neurological integration through meaningful sensory–motor activities. Its evidence base has evolved from early observational studies [29] to support from multiple randomized controlled trials, which confirm its efficacy in improving self-care, social function, school performance, and daily living skills in children with ASD [18,30,31]. Systematic reviews also affirm its positive effects [6,32]. The current research frontier involves employing multimodal assessments to elucidate its neural mechanisms. For instance, studies utilizing functional near-infrared spectroscopy and electroencephalography have revealed that training can enhance prefrontal activation and improve neural responses to face processing, thereby linking behavioral improvements to changes in brain function [33,34]. Simultaneously, research is increasingly focusing on the postural control deficits in individuals with ASD and their intrinsic link with sensory integration difficulties [35,36]. This has led to the development of targeted motor-based interventions, such as square-stepping exercise and rock climbing, aimed at enhancing sensory–motor integration and overall function [37,38]. Behavioral and developmental interventions constitute the cornerstone of the ASD intervention framework. The research focus has shifted from validating efficacy to exploring how to implement these interventions more efficiently, with greater specificity, and with enhanced feasibility [9]. Long-term studies of Early Intensive Behavioral Intervention have demonstrated significant improvements in cognition, language, and adaptive skills for some children [39], and systematic reviews support its benefits compared to routine care [40]. However, the therapeutic effects exhibit significant individual variability, which is strongly associated with factors such as age at intervention onset, baseline cognitive level, and symptom severity [41,42]. Furthermore, the long-term benefits may not be fully sustained [43]. This has driven the evolution of practice toward more flexible and personalized models. Concurrently, Naturalistic Developmental Behavioral Interventions have rapidly gained prominence. These approaches emphasize teaching within everyday contexts by leveraging the child’s motivations. For instance, the Early Start Denver Model has been confirmed by randomized controlled trials to improve cognitive ability, adaptive behavior, and neural responses in young children [8,44], and to effectively enhance joint attention and social initiation skills [45,46]. To enhance the accessibility and sustainability of interventions, parent training and remote service delivery models have been significantly developed. Studies indicate that training parents can effectively improve children’s skills and alleviate parental stress [47,48]. Furthermore, the remote implementation of behavioral interventions and training has been demonstrated to be both feasible and effective, greatly expanding the reach of services [49,50,51,52,53]. Moreover, the principles of behavioral intervention are widely applied to teach specific skills (such as theory of mind) and to manage comorbid conditions (e.g., anxiety, sleep disturbances), demonstrating their broad applicability in addressing the diverse needs of individuals with ASD [54,55,56,57,58]. Looking ahead, future large-scale pragmatic randomized controlled trials will further evaluate their effectiveness in real-world settings [59], marking the field’s continued evolution toward more precise, mechanism-informed, and scalable directions.

The third pathway highlights the socio-emotional dimension of art and animal-assisted therapies. Beyond classic sensory integration therapy, other intervention formats that target the sensory and motor systems have also been widely investigated. Bibliometric data indicate that although therapies such as “music therapy” (15 occurrences, 2012), “animal-assisted therapy” (8 occurrences, 2007), and “art therapy” (4 occurrences, 2022) appear with relatively low frequency in the literature, they receive widespread attention from parents and practitioners. However, this area is characterized by a notable contradiction between widespread use and the lack of high-quality evidence. Art and animal-assisted therapies represent distinctive branches within the non-pharmacological interventions for ASD. Through non-verbal and affective media such as music, visual art, drama, and animals, they offer individuals with ASD unique avenues for social interaction and emotional expression [60,61]. Among these approaches, music therapy has been the most extensively studied. An updated Cochrane systematic review indicates that it may lead to improvements in overall state, quality of life, and symptom severity [62]. Multiple randomized controlled trials and meta-analyses also support its positive effects in enhancing social communication, language, and behavioral symptoms [63,64,65]. However, the large-scale randomized controlled trial “TIME-A” yielded mixed findings, suggesting that its efficacy may be influenced by factors such as intervention format, intensity, and outcome measures [5,66]. Mechanistic research is deepening, revealing that musical synchrony, shared engagement, and evoked movement within therapy sessions constitute important active components. Such interventions have also been found to enhance connectivity in brain auditory–motor pathways, providing a neural basis for the facilitation of social interaction [67,68]. Other art-based therapies such as visual art therapy and drama therapy have also shown potential in improving social responsiveness and adaptive skills [69,70]. However, high-quality randomized controlled evidence remains relatively limited, and the details of intervention design are crucial [71]. Animal-assisted therapy (e.g., dog- or horse-assisted) has been widely reported in observational and some quantitative studies to promote social engagement, increase positive behaviors, and reduce anxiety [72,73,74]. The underlying mechanism likely stems from the animal’s role as a “social catalyst,” which attracts and prompts interactions [75]. Yet research in this area is often limited by factors such as small sample sizes and heterogeneous study designs. Systematic reviews, while acknowledging the encouraging preliminary evidence, emphasize the need for more rigorous studies to establish its evidence-based status [76,77]. Overall, these therapies highlight the value of affective and relational dimensions in ASD intervention. That said, future efforts require more standardized, mechanism-oriented research to strengthen their scientific foundation and determine the most effective practical formats.

Non-pharmacological interventions for autism spectrum disorder should not be viewed as isolated approaches. Future intervention research should actively explore the synergistic effects of combining multiple methods—for example, integrating neuromodulation with behavioral training, or combining physical activity interventions with sensory integration principles. Such integration is expected to produce a synergistic effect where the whole is greater than the sum of its parts. At the same time, attention must be given to technology-driven interventions, such as artificial intelligence-assisted social skills training and telehealth platforms, to enhance the accessibility and scalability of interventions.

This study has several limitations. First, the literature data were sourced exclusively from the Web of Science Core Collection. Although this database provides quality-controlled coverage of high-impact international English-language journals and a well-established citation indexing system, offering standardized data suitable for bibliometric analysis and aligning with the objective of capturing core research in the field of non-pharmacological interventions for ASD, reliance on a single database introduces inherent bias. Compared with Scopus, the Web of Science Core Collection has narrower coverage of emerging journals and key journals from non-English-speaking regions, which may result in the omission of important studies from Europe, Asia, and other areas. This may lead to an incomplete picture of the global distribution of research output and collaboration networks, and may particularly underestimate the contributions of countries with moderate research output. Meanwhile, PubMed, as a specialized database in the biomedical domain, offers more comprehensive coverage of clinical and intervention trial literature, with indexing dimensions better aligned with medical research characteristics. Its exclusion may result in the omission of original studies focusing on clinical applications and small-sample intervention trials in ASD non-pharmacological treatment, thereby affecting the completeness of the depiction of clinical research hotspots and intervention practices in the field. Moreover, differences in indexing rules and keyword extraction systems across databases may introduce subtle inaccuracies in the results of keyword co-occurrence, clustering, and burst detection analyses. To mitigate such limitations, future research may consider integrating multi-source data from databases such as Scopus, PubMed, and PsycINFO to conduct comparative analyses and further validate the robustness of the findings. Secondly, this study does not adequately address the heterogeneity of research findings in the field of neuromodulation. Third, the selection and standardization of keywords may influence the precise interpretation of the clustering results.

## 5. Conclusions

This study represents the first systematic bibliometric analysis of the non-pharmacological intervention field for ASD spanning 2001 to 2025, delineating its clear trajectory from an initial nascent phase, through a period of fluctuating growth, to its recent vigorous development. The findings indicate that annual publication output entered a phase of rapid growth starting in 2020, signifying the field’s emergence as a prominent international academic frontier. Global collaboration exhibits a “core–hub” network structure, with the United States and China serving as the primary knowledge production cores and countries such as Spain and Australia acting as critical connecting hubs, efficiently driving transnational knowledge flow and integration. Furthermore, keyword evolution analysis details a fundamental paradigm shift within the field—from an early focus on diagnostic concepts and behavioral interventions toward contemporary research frontiers centered on neuromodulation techniques, brain network mechanisms, and evidence-based precision approaches. These findings, objectively presented through visual mappings, portray the field’s developmental landscape and dynamic structure, providing an empirical basis for the academic community to comprehend its evolutionary patterns and plan future collaborations. Looking ahead, key directions for the field’s sustained advancement will include deepening integrated research on neural mechanisms and behavioral interventions, developing individualized precision protocols, and promoting the translation of interdisciplinary findings into accessible clinical practice.

## Figures and Tables

**Table 1:** Publication Count and Centrality Metrics of Core Countries/Regions.

Frequency	Centrality	Year	Country
1305	0.23	2001	USA
389	0.02	2004	PEOPLES R CHINA
322	0.17	2001	ENGLAND
282	0.05	2001	ITALY
255	0.04	2004	CANADA
250	0.14	2003	AUSTRALIA
125	0.07	2001	FRANCE
122	0.04	2005	GERMANY
115	0.01	2005	JAPAN
98	0.04	2006	SPAIN

**Table 2:** Publication Count of Core Institutions in the Field of ASD Non-Pharmacological Intervention Research.

Frequency	Centrality	Year	Institution
200	0.02	2001	University of California System
129	0.08	2002	University of London
117	0.01	2006	Harvard University
98	0.03	2006	Harvard University Medical Affiliates
98	0.04	2002	University of California Los Angeles
90	0.02	2011	University of Toronto
79	0.02	2008	Harvard Medical School
75	0.03	2002	King’s College London
65	0.09	2010	University of North Carolina
63	0.02	2010	University of Washington
63	0.02	2010	University of Washington Seattle
55	0.08	2010	Centre National de la Recherche Scientifique (CNRS)
53	0.02	2011	University System of Ohio
51	0.02	2001	Institut National de la Sante et de la Recherche Medicale (Inserm)

**Table 3:** Distribution of Highly Cited Publications.

Frequency	Year	Publications
151	2014	Carter MJ, 2014, THER RECREAT J, V48, P275
61	2018	Baio J, 2018, MMWR SURVEILL SUMM, V67, P1, DOI 10.15585/mmwr.ss6706a1
60	2022	Zeidan J, 2022, AUTISM RES, V15, P778, DOI 10.1002/aur.2696
59	2023	Maenner MJ, 2023, MMWR SURVEILL SUMM, V72, P1, DOI 10.15585/mmwr.ss7202a1
46	2016	Sparrow SS, 2016, VINELAND ADAPTIVE BEHAVIOR SCALES, V0, P0, DOI 10.1177/0829573517733845
46	2020	Sandbank M, 2020, PSYCHOL BULL, V146, P1, DOI 10.1037/bul0000215
44	2018	Lord C, 2018, LANCET, V392, P508, DOI 10.1016/S0140-6736(18)31129-2
44	2021	Maenner MJ, 2021, MMWR SURVEILL SUMM, V70, P0, DOI 10.15585/mmwr.ss7011a1
43	2020	Lord C, 2020, NAT REV DIS PRIMERS, V6, P0, DOI 10.1038/s41572-019-0138-4
39	2015	Schreibman L, 2015, J AUTISM DEV DISORD, V45, P2411, DOI 10.1007/s10803-015-2407-8

**Table 4:** Distribution of Highly Cited Journals.

Frequency	Year	Journals
2709	2001	J AUTISM DEV DISORD
1561	2003	AUTISM
1434	2001	J CHILD PSYCHOL PSYC
1257	2010	AUTISM RES
1151	2009	RES AUTISM SPECT DIS
1082	2009	PLOS ONE
991	2001	J AM ACAD CHILD PSY
975	2001	PEDIATRICS
951	2005	RES DEV DISABIL
731	2003	NEUROSCI BIOBEHAV R

**Table 5:** High-Frequency Keywords and Their Centrality Metrics in ASD Non-Pharmacological Intervention Research.

Count	Centrality	Year	Keyword
955	0.03	2001	children
899	0.03	2003	autism spectrum disorder
509	0.02	2001	joint attention
443	0.06	2002	autism
442	0.01	2003	young children
402	0.03	2005	adolescents
313	0.02	2005	spectrum disorder
296	0.02	2005	individuals
286	0.02	2006	autism spectrum disorders
240	0.03	2006	spectrum disorders
236	0.02	2005	social skills
236	0.01	2003	intervention
235	0.01	2011	physical activity
222	0.05	2001	adults
190	0.01	2005	prevalence
186	0.02	2003	language
172	0.03	2002	brain
169	0.04	2004	asperger syndrome
166	0.05	2002	behavior
163	0.02	2005	skills
148	0.03	2005	high functioning autism
148	0.02	2007	communication
137	0.01	2008	social cognition
132	0.02	2010	interventions
125	0.03	2002	infants

## Data Availability

The data supporting the findings of this study were obtained from the Web of Science Core Collection, a publicly available database. The datasets analyzed during the current study are available from the corresponding author upon reasonable request.

## Abbreviations

The following abbreviations are used in this manuscript:

ASD	Autism Spectrum Disorder
rTMS	repetitive Transcranial Magnetic Stimulation
tDCS	transcranial Direct Current Stimulation
RCT	Randomized Controlled Trial
